# Supplementary material for: Molecular tuning of sea anemone stinging
Source: eLife. 2023 Oct 31;12:RP88900. doi: 10.7554/eLife.88900 (PMC10617991; doi:10.7554/eLife.88900)
Supplement: Figure 5—source data 1. [file elife-88900-fig5-data1.docx]

**Figure 5 – source data 1: Wild type and Chimeric Ca_V_β amino acid sequences.**

| Protein name | Amino Acid Sequence |
| --- | --- |
| *Exaiptasia diaphana*  Ca_V_β1 (EdCa_V_β1) | MAQDFALSNRDIELDSLEHDSTGSSTPSEIQRWHMYSDRSGRVVCKDSEPAYRASDTSSVDEDKETSRRELERRAWEALQAARSKPVAFAVRTNIAYEGSEDDDSPVHGAAVSFNVKDFLHVKEKFNDDWWIGRVVKEGCDIGFIPTPSKLKSLQQVGPATGGRPVRGSSKTVFHFNDMVNQAQSPTNTSPSRHSSASVDAENGMEYNEEEQHSPTSPTSKTSTLPRSASGNTVTSQSAPGQQGKSKKAFFKKQEQLPPYDVVPSMRPIVLVGPSLKGYEVTDMMQKALFDYMKHQFSGRVLISRVTSDISLAKRSNLANPSKRNIIERSNSKNSGLAEVQQEIERIFELSRGLNLVVLDCDTVNHPTQLAKTSLAPLVVYVKISAPKVLQRLIKTRGKTQSRALNVQLVAAEKLAQCSEDLYDLILDETQLQDACHHLGEFLESYWRATHPPNQPGSRPPNMQQSTPQYNVIEAGERPSVYL |
| *Exaiptasia diaphana*  Ca_V_β2 (EdCa_V_β2) | MGNTDSVQSFTKDSEPAYRASDTSSVDEDKETSRRELERRAWEALQAARSKPVAFAVRTNIAYEGSEDDDSPVHGAAVSFNVKDFLHVKEKFNDDWWIGRVVKEGCDIGFIPTPSKLKSLQQVGPATGGRPVRGSSKTVFHFNDMVNQAQSPTNTSPSRHSSASVVDAENGMEYNEEEQHSPTSPTSKTSTLPRSASGNTVTSQSAPGQQGKSKKAFFKKQEQLPPYDVVPSMRPIVLVGPSLKGYEVTDMMQKALFDYMKHQFSGRVLISRVTSDISLAKRSNLANPSKRNIIERSNSKNSGLAEVQQEIERIFELSRGLNLVVLDCDTVNHPTQLAKTSLAPLVVYVKISAPKVLQRLIKTRGKTQSRALNVQLVAAEKLAQCSEDLYDLILDETQLQDACHHLGEFLESYWRATHPPNQPGSRPPNMQQSTPQYNVIEAGERPSVYL |
| *Cyanea*  *Capillata* Ca_V_β  (CcCa_V_β) | MWFGTKKSKDSERRKRQPIDVYREQALSVNPAYIWGDDLDSRKTSGTSSEYGEDDIEQIRVQALEQLAAARVKPVAFAMRANYGYNGAEDDDSPIHGMALSFEPKDFLHIKEKFNNDWLIGRVVREGCDIGFIPSPSKLESLRLSGLAGRKMRQSSTSSNLHLQDAFSASSPSEDRQNSFDDESLPPSSPVKSVNPGVIGQPNSKTAKKGIFKKNDSLPPYDVVPSMRPVIFVGPSLKGYEVTDMMQKALFDYLKHRFQGRIVITRVTADISTAKKSTIQNLAKKPIIKERGATQASQEVNQEIERIFELCRNLQLVVLDSYTVNYPAQVAKTSLAPIIVYIKISSPKVLTRLVKSRGKSQSKNLNVQLVAAVKLGQCSEDMYDVVLDETQLEDACEHLGEFLEAYWRAAHPSQSNFGAAGAPGSFTANGQPVVVNYNSMDPFSAQSPTRHLRTAQV |
| *Physalia physalis* Ca_V_β (PpCa_V_β) | MVTASYNVPLDNTSATHSFNYPHAFLLTHSSCSYHSNEGFINSSTEVDIVDENDFKPLFEGNSNEPHCQKKVISFSSLLDNVVAPIWYFFEMGDEFDSRKTSGTSSEYGEEDVEALRVQALEQLAAAASKPVAFAVRANYGYNGSEDEDCPVNGMAVSFEAKDCLHIKVKFNNDWWIGRVVKEGHDIGFIPSASRLDNIRQSGISGKLKLRQSSTSSNMNLEDQSQPLSREQDNRSPSEERGTSFDDDSPASPLRNPSGSSLTANNNNNNSNTASNVNNSQPKGKKGIFKKSENLPPYDVVPSMRPIIFVGPSLKGYEVTNMMQKALFDYLKHRFQGRIVITRVGADISLAKRSAFQHPGKQPVIQKKGNTQSGIVEVQQEIERIFELCRSMQLVVLDCESINHPSQVAKTSLAPIIAMIRIASPKVLTRLIKSRGKSQTKHLNFQLVAAEKLNQCTEDMFDVILDENQLEDACEHLGDFLEAYWRSAVPPRRPYVNSDNRSYNNAGGQSIGNYNGGGQYNGTPQRHLRTAQV |
| *Cassiopea xamachana* Ca_V_β (CxCa_V_β) | MVQKSGMSRGPYPPSQEIPMEVFDPSPQGKYSKRKGRFKRSDGSTSSDTTSNSFVRQGSAESYTSRPSDSDVSLEEDREALRKEAERQALAQLEKAKTKPVAFAVRTNVGYNPSPGDEVPVQGVAITFEPKDFLHIKEKYNNDWWIGRLVKEGCEVGFIPSPVKLDSLRLLQEQTLRQNRLSSSKSGDNSSSSLGDVVTGTRRPTPPASAKQKQKSTEHVPPYDVVPSMRPIILVGPSLKGYEVTDMMQKALFDFLKHRFDGRISITRVTADISLAKRSVLNNPSKHIIIERSNTRSSLAEVQSEIERIFELARTLQLVALDADTINHPAQLSKTSLAPIIVYIKITSPKVLQRLIKSRGKSQSKHLNVQIAASEKLAQCPPEMFDIILDENQLEDACEHLAEYLEAYWKATHPPSSTPPNPLLNRTMATAALAASPAPVSNLQGPYLASGDQPLDRATGEHASVHEYPGELGQPPGLYPSNHPPGRAGTLRALSRQDTFDADTPGSRNSAYTEPGDSCVDMETDPSEGPGPGDPAGGGTPPARQGSWEEEEDYEEEMTDNRNRGRNKARYCAEGGGPVLGRNKNELEGWGQGVYIR |
| *Clytia hemisphaerica* Ca_V_β (ChCa_V_β) | MMHGSQTEPAISSMTSERNHKNLSHGSRTSINSQRSTNKKVNSHVSFDESTAAPSSKKPGALSAAGGKKSVDDNFSSSVLQTVFALRWQKKAAQKKKKPDDFQQMYMHSMSGALGSIIGDEFDGRKTSGTSSEYGDGEDLEALRILALEKLQAARTRPVAFAVRANYGYNGSEDDDSPVHGMAVSFEKDDCLHIKDKFNKDWWIGRVVKEGHNIGFVPSPDKLESIRQSGVSGKLKMRQSSTSSNMNLHDDPQNQRSPLGEAGGNNSFDDETVNSPVRNVSTESNNTNNNNTTNSLNAQKGKKGIFKKNEQLHPYYVIPSMRPIIFVGPSLKGYEVTDMMQKALFDYLKHRFSERIIFTRVNADISLAKRSNLNNQNRQPNFPKKSNGQAGLAEVQEEVNRIFELCRSSQLVVLDCDTINNPSQVIKTSLAPIIVAIKIASPKVLTRLIKSRGKNQVKHLNIQMIAADKLSQCNEEMFDVVLDENQLEDACEHLGEFLEAYWRAAVPGAQEGLISQENGGFVNQGGPNGAGYNGVDQYGTPQRNLRTAQV |
| *Nematostella vectensis*  cacnb2.1  (NVE β) | MEPEPGLSEQDIELDSLEQVSTASSFHSDIQRHYNDGREASRFIGADDFNRDSDPAYRASDTSSIEEDRETSRRELERRAWDALQAARSKPVAFAVRTNLRYDGSEDDDSPVHGAAVSFEAKDFLHVKEKFNDDWWIGRVVKEGCDIGFIPTPSKLKSLQQIGGTASGRGMRNSKRDVFQFDMVNQAQSPTNTSPSRHSSTSVDAENGVEYDDDQQSPTSPTNKTLPRSASGTTVSSQPGTATGTQGKPKKGLFKKQEQLPPYDVVPSMRPIVLVGPSLKGYEVTDMMQKALLDFMKHRFSGRVLIARVTSDISLAKRTNMSNPGKQTIMERTKNKNTGLAEVQQEIERIFELARGLNLVVLDCETVNHPTQLAKTSLAPMIVYIKIAAPKVLQRLIKTRGKSQSRNLSIQLVAAEKLAQCSEDMYDLVLEETQLDDACEHLGEFLESYWRATHPPNQPGSRPPNVQPSNSTPQYNVIEGGERPSVYL |
| Rat cacnb2a  (Rat β) | MQCCGLVHRRRVRVSYGSADSYTSRPSDSDVSLEEDREAVRREAERQAQAQLEKAKTKPVAFAVRTNVRYSAAQEDDVPVPGMAISFEAKDFLHVKEKFNNDWWIGRLVKEGCEIGFIPSPVKLENMRLQHEQRAKQGKFYSSKSGGNSSSSLGDIVPSSRKSTPPSSAIDIDATGLDAEENDIPANHRSPKPSANSVTSPHSKEKRMPFFKKTEHTPPYDVVPSMRPVVLVGPSLKGYEVTDMMQKALFDFLKHRFEGRISITRVTADISLAKRSVLNNPSKHAIIERSNTRSSLAEVQSEIERIFELARTLQLVVLDADTINHPAQLSKTSLAPIIVYVKISSPKVLQRLIKSRGKSQAKHLNVQMVAADKLAQCPPQESFDVILDENQLEDACEHLADYLEAYWKATHPPSSNLPNPLLSRTLATSTLPLSPTLASNSQGSQGDQRTDRSAPRSASQAEEEPCLEPVKKSQHRSSSATHQNHRSGTGRGLSRQETFDSETQESRDSAYVEPKEDYSHEHVDRYVPHREHNHREESHSSNGHRHREPRHRTRDMGRDQDHNECSKQRSRHKSKDRYCDKEGEVISKRRSEAGEWNRDVYIRQ |
| Rat β with  NVE Hook | MQCCGLVHRRRVRVSYGSADSYTSRPSDSDVSLEEDREAVRREAERQAQAQLEKAKTKPVAFAVRTNVRYSAAQEDDVPVPGMAISFEAKDFLHVKEKFNNDWWIGRLVKEGCEIGFIPSPSKLKSLQQIGGTASGRGMRNSKRDVFQFDMVNQAQSPTNTSPSRHSSTSVDAENGVEYDDDQQSPTSPTNKTLPRSASGTTVSSQPGTATGTQGKPKKGLFKKQEQLPPYDVVPSMRPVVLVGPSLKGYEVTDMMQKALFDFLKHRFEGRISITRVTADISLAKRSVLNNPSKHAIIERSNTRSSLAEVQSEIERIFELARTLQLVVLDADTINHPAQLSKTSLAPIIVYVKISSPKVLQRLIKSRGKSQAKHLNVQMVAADKLAQCPPQESFDVILDENQLEDACEHLADYLEAYWKATHPPSSNLPNPLLSRTLATSTLPLSPTLASNSQGSQGDQRTDRSAPRSASQAEEEPCLEPVKKSQHRSSSATHQNHRSGTGRGLSRQETFDSETQESRDSAYVEPKEDYSHEHVDRYVPHREHNHREESHSSNGHRHREPRHRTRDMGRDQDHNECSKQRSRHKSKDRYCDKEGEVISKRRSEAGEWNRDVYIRQ |
| NVE β with  Rat Hook | MEPEPGLSEQDIELDSLEQVSTASSFHSDIQRHYNDGREASRFIGADDFNRDSDPAYRASDTSSIEEDRETSRRELERRAWDALQAARSKPVAFAVRTNLRYDGSEDDDSPVHGAAVSFEAKDFLHVKEKFNDDWWIGRVVKEGCDIGFIPTPVKLENMRLQHEQRAKQGKFYSSKSGGNSSSSLGDIVPSSRKSTPPSSAIDIDATGLDAEENDIPANHRSPKPSANSVTSPHSKEKRMPFFKKTEHTPPYDVVPSMRPIVLVGPSLKGYEVTDMMQKALLDFMKHRFSGRVLIARVTSDISLAKRTNMSNPGKQTIMERTKNKNTGLAEVQQEIERIFELARGLNLVVLDCETVNHPTQLAKTSLAPMIVYIKIAAPKVLQRLIKTRGKSQSRNLSIQLVAAEKLAQCSEDMYDLVLEETQLDDACEHLGEFLESYWRATHPPNQPGSRPPNVQPSNSTPQYNVIEGGERPSVYL |
| Rat β with  NVE GK  domain | MQCCGLVHRRRVRVSYGSADSYTSRPSDSDVSLEEDREAVRREAERQAQAQLEKAKTKPVAFAVRTNVRYSAAQEDDVPVPGMAISFEAKDFLHVKEKFNNDWWIGRLVKEGCEIGFIPSPVKLENMRLQHEQRAKQGKFYSSKSGGNSSSSLGDIVPSSRKSTPPSSAIDIDATGLDAEENDIPANHRSPKPSANSVTSPHSKEKRMPFFKKTEHTPPYDVVPSMRPIVLVGPSLKGYEVTDMMQKALLDFMKHRFSGRVLIARVTSDISLAKRTNMSNPGKQTIMERTKNKNTGLAEVQQEIERIFELARGLNLVVLDCETVNHPTQLAKTSLAPMIVYIKIAAPKVLQRLIKTRGKSQSRNLSIQLVAAEKLAQCSEDMYDLVLEETQLDDACEHLGEFLESYWRATHPPNQPGSRPPNVQPSNSTPQYNVIEGGERPSVYL |
| NVE β with  Rat GK  domain | MEPEPGLSEQDIELDSLEQVSTASSFHSDIQRHYNDGREASRFIGADDFNRDSDPAYRASDTSSIEEDRETSRRELERRAWDALQAARSKPVAFAVRTNLRYDGSEDDDSPVHGAAVSFEAKDFLHVKEKFNDDWWIGRVVKEGCDIGFIPTPSKLKSLQQIGGTASGRGMRNSKRDVFQFDMVNQAQSPTNTSPSRHSSTSVDAENGVEYDDDQQSPTSPTNKTLPRSASGTTVSSQPGTATGTQGKPKKGLFKKQEQLPPYDVVPSMRPVVLVGPSLKGYEVTDMMQKALFDFLKHRFEGRISITRVTADISLAKRSVLNNPSKHAIIERSNTRSSLAEVQSEIERIFELARTLQLVVLDADTINHPAQLSKTSLAPIIVYVKISSPKVLQRLIKSRGKSQAKHLNVQMVAADKLAQCPPQESFDVILDENQLEDACEHLADYLEAYWKATHPPSSNLPNPLLSRTLATSTLPLSPTLASNSQGSQGDQRTDRSAPRSASQAEEEPCLEPVKKSQHRSSSATHQNHRSGTGRGLSRQETFDSETQESRDSAYVEPKEDYSHEHVDRYVPHREHNHREESHSSNGHRHREPRHRTRDMGRDQDHNECSKQRSRHKSKDRYCDKEGEVISKRRSEAGEWNRDVYIRQ |
| Rat 5’ on  NVE β | MQCCGLVHRRRVRVSYGSADSYTSRPSDSDVSLEEDREAVRREAERQAQAQLEKAKTKPVAFAVRTNLRYDGSEDDDSPVHGAAVSFEAKDFLHVKEKFNDDWWIGRVVKEGCDIGFIPTPSKLKSLQQIGGTASGRGMRNSKRDVFQFDMVNQAQSPTNTSPSRHSSTSVDAENGVEYDDDQQSPTSPTNKTLPRSASGTTVSSQPGTATGTQGKPKKGLFKKQEQLPPYDVVPSMRPIVLVGPSLKGYEVTDMMQKALLDFMKHRFSGRVLIARVTSDISLAKRTNMSNPGKQTIMERTKNKNTGLAEVQQEIERIFELARGLNLVVLDCETVNHPTQLAKTSLAPMIVYIKIAAPKVLQRLIKTRGKSQSRNLSIQLVAAEKLAQCSEDMYDLVLEETQLDDACEHLGEFLESYWRATHPPNQPGSRPPNVQPSNSTPQYNVIEGGERPSVYL |
| Rat 5’ + SH3  on NVE β | MQCCGLVHRRRVRVSYGSADSYTSRPSDSDVSLEEDREAVRREAERQAQAQLEKAKTKPVAFAVRTNVRYSAAQEDDVPVPGMAISFEAKDFLHVKEKFNNDWWIGRLVKEGCEIGFIPSPSKLKSLQQIGGTASGRGMRNSKRDVFQFDMVNQAQSPTNTSPSRHSSTSVDAENGVEYDDDQQSPTSPTNKTLPRSASGTTVSSQPGTATGTQGKPKKGLFKKQEQLPPYDVVPSMRPIVLVGPSLKGYEVTDMMQKALLDFMKHRFSGRVLIARVTSDISLAKRTNMSNPGKQTIMERTKNKNTGLAEVQQEIERIFELARGLNLVVLDCETVNHPTQLAKTSLAPMIVYIKIAAPKVLQRLIKTRGKSQSRNLSIQLVAAEKLAQCSEDMYDLVLEETQLDDACEHLGEFLESYWRATHPPNQPGSRPPNVQPSNSTPQYNVIEGGERPSVYL |
| NVE 5’ +  SH3 on Rat β | MEPEPGLSEQDIELDSLEQVSTASSFHSDIQRHYNDGREASRFIGADDFNRDSDPAYRASDTSSIEEDRETSRRELERRAWDALQAARSKPVAFAVRTNVRYSAAQEDDVPVPGMAISFEAKDFLHVKEKFNNDWWIGRLVKEGCEIGFIPSPVKLENMRLQHEQRAKQGKFYSSKSGGNSSSSLGDIVPSSRKSTPPSSAIDIDATGLDAEENDIPANHRSPKPSANSVTSPHSKEKRMPFFKKTEHTPPYDVVPSMRPVVLVGPSLKGYEVTDMMQKALFDFLKHRFEGRISITRVTADISLAKRSVLNNPSKHAIIERSNTRSSLAEVQSEIERIFELARTLQLVVLDADTINHPAQLSKTSLAPIIVYVKISSPKVLQRLIKSRGKSQAKHLNVQMVAADKLAQCPPQESFDVILDENQLEDACEHLADYLEAYWKATHPPSSNLPNPLLSRTLATSTLPLSPTLASNSQGSQGDQRTDRSAPRSASQAEEEPCLEPVKKSQHRSSSATHQNHRSGTGRGLSRQETFDSETQESRDSAYVEPKEDYSHEHVDRYVPHREHNHREESHSSNGHRHREPRHRTRDMGRDQDHNECSKQRSRHKSKDRYCDKEGEVISKRRSEAGEWNRDVYIRQ |
| NVE 5’ on  Rat β | MEPEPGLSEQDIELDSLEQVSTASSFHSDIQRHYNDGREASRFIGADDFNRDSDPAYRASDTSSIEEDRETSRRELERRAWDALQAARSKPVAFAVRTNLRYDGSEDDDSPVHGAAVSFEAKDFLHVKEKFNDDWWIGRVVKEGCDIGFIPTPVKLENMRLQHEQRAKQGKFYSSKSGGNSSSSLGDIVPSSRKSTPPSSAIDIDATGLDAEENDIPANHRSPKPSANSVTSPHSKEKRMPFFKKTEHTPPYDVVPSMRPVVLVGPSLKGYEVTDMMQKALFDFLKHRF  EGRISITRVTADISLAKRSVLNNPSKHAIIERSNTRSSLAEVQSEIERIFELARTLQLVVLDADTINHPAQLSKTSLAPIIVYVKISSPKVLQRLIKSRGKSQAKHLNVQMVAADKLAQCPPQESFDVILDENQLEDACEHLADYLEAYWKATHPPSSNLPNPLLSRTLATSTLPLSPTLASNSQGSQGDQRTDRSAPRSASQAEEEPCLEPVKKSQHRSSSATHQNHRSGTGRGLSRQETFDSETQESRDSAYVEPKEDYSHEHVDRYVPHREHNHREESHSSNGHRHREPRHRTRDMGRDQDHNECSKQRSRHKSKDRYCDKEGEVISKRRSEAGEWNRDVYIRQ |
| EdCa_V_β2 with NVE β NTerm | MEPEPGLSEQDIELDSLEQVSTASSFHSDIQRHYNDGREASRFIGADDFNRDSDPAYRASDTSSIEEDRETSRRELERRAWDALQAARSKPVAFAVRTNLRYDGSEDDDSPVHGAAVSFEAKDFLHVKEKFNDDWWIGRVVKEGCDIGFIPTPSKLKSLQQVGPATGGRPVRGSSKTVFHFNDMVNQAQSPTNTSPSRHSSASVVDAENGMEYNEEEQHSPTSPTSKTSTLPRSASGNTVTSQSAPGQQGKSKKAFFKKQEQLPPYDVVPSMRPIVLVGPSLKGYEVTDMMQKALFDYMKHQFSGRVLISRVTSDISLAKRSNLANPSKRNIIERSNSKNSGLAEVQQEIERIFELSRGLNLVVLDCDTVNHPTQLAKTSLAPLVVYVKISAPKVLQRLIKTRGKTQSRALNVQLVAAEKLAQCSEDLYDLILDETQLQDACHHLGEFLESYWRATHPPNQPGSRPPNMQQSTPQYNVIEAGERPSVYL |
| EdCa_V_β2 with CcCa_V_β NTerm | MWFGTKKSKDSERRKRQPIDVYREQALSVNPAYIWGDDLDSRKTSGTSSEYGEDDIEQIRVQALEQLAAARVKPVAFAMRANYGYNGAEDDDSPIHGMALSFEPKDFLHIKEKFNNDWLIGRVVREGCDIGFIPSPSKLKSLQQVGPATGGRPVRGSSKTVFHFNDMVNQAQSPTNTSPSRHSSASVVDAENGMEYNEEEQHSPTSPTSKTSTLPRSASGNTVTSQSAPGQQGKSKKAFFKKQEQLPPYDVVPSMRPIVLVGPSLKGYEVTDMMQKALFDYMKHQFSGRVLISRVTSDISLAKRSNLANPSKRNIIERSNSKNSGLAEVQQEIERIFELSRGLNLVVLDCDTVNHPTQLAKTSLAPLVVYVKISAPKVLQRLIKTRGKTQSRALNVQLVAAEKLAQCSEDLYDLILDETQLQDACHHLGEFLESYWRATHPPNQPGSRPPNMQQSTPQYNVIEAGERPSVYL |
| EdCa_V_β2 with PpCa_V_β NTerm | MVTASYNVPLDNTSATHSFNYPHAFLLTHSSCSYHSNEGFINSSTEVDIVDENDFKPLFEGNSNEPHCQKKVISFSSLLDNVVAPIWYFFEMGDEFDSRKTSGTSSEYGEEDVEALRVQALEQLAAAASKPVAFAVRANYGYNGSEDEDCPVNGMAVSFEAKDCLHIKVKFNNDWWIGRVVKEGHDIGFIPSPSKLKSLQQVGPATGGRPVRGSSKTVFHFNDMVNQAQSPTNTSPSRHSSASVVDAENGMEYNEEEQHSPTSPTSKTSTLPRSASGNTVTSQSAPGQQGKSKKAFFKKQEQLPPYDVVPSMRPIVLVGPSLKGYEVTDMMQKALFDYMKHQFSGRVLISRVTSDISLAKRSNLANPSKRNIIERSNSKNSGLAEVQQEIERIFELSRGLNLVVLDCDTVNHPTQLAKTSLAPLVVYVKISAPKVLQRLIKTRGKTQSRALNVQLVAAEKLAQCSEDLYDLILDETQLQDACHHLGEFLESYWRATHPPNQPGSRPPNMQQSTPQYNVIEAGERPSVYL |
| EdCa_V_β2 with Rat β NTerm | MQCCGLVHRRRVRVSYGSADSYTSRPSDSDVSLEEDREAVRREAERQAQAQLEKAKTKPVAFAVRTNVRYSAAQEDDVPVPGMAISFEAKDFLHVKEKFNNDWWIGRLVKEGCEIGFIPSPSKLKSLQQVGPATGGRPVRGSSKTVFHFNDMVNQAQSPTNTSPSRHSSASVVDAENGMEYNEEEQHSPTSPTSKTSTLPRSASGNTVTSQSAPGQQGKSKKAFFKKQEQLPPYDVVPSMRPIVLVGPSLKGYEVTDMMQKALFDYMKHQFSGRVLISRVTSDISLAKRSNLANPSKRNIIERSNSKNSGLAEVQQEIERIFELSRGLNLVVLDCDTVNHPTQLAKTSLAPLVVYVKISAPKVLQRLIKTRGKTQSRALNVQLVAAEKLAQCSEDLYDLILDETQLQDACHHLGEFLESYWRATHPPNQPGSRPPNMQQSTPQYNVIEAGERPSVYL |
| EdCa_V_β2 with CxCa_V_β NTerm | MVQKSGMSRGPYPPSQEIPMEVFDPSPQGKYSKRKGRFKRSDGSTSSDTTSNSFVRQGSAESYTSRPSDSDVSLEEDREALRKEAERQALAQLEKAKTKPVAFAVRTNVGYNPSPGDEVPVQGVAITFEPKDFLHIKEKYNNDWWIGRLVKEGCEVGFIPSPSKLKSLQQVGPATGGRPVRGSSKTVFHFNDMVNQAQSPTNTSPSRHSSASVVDAENGMEYNEEEQHSPTSPTSKTSTLPRSASGNTVTSQSAPGQQGKSKKAFFKKQEQLPPYDVVPSMRPIVLVGPSLKGYEVTDMMQKALFDYMKHQFSGRVLISRVTSDISLAKRSNLANPSKRNIIERSNSKNSGLAEVQQEIERIFELSRGLNLVVLDCDTVNHPTQLAKTSLAPLVVYVKISAPKVLQRLIKTRGKTQSRALNVQLVAAEKLAQCSEDLYDLILDETQLQDACHHLGEFLESYWRATHPPNQPGSRPPNMQQSTPQYNVIEAGERPSVYL |
| EdCa_V_β2 with ChCa_V_β NTerm | MMHGSQTEPAISSMTSERNHKNLSHGSRTSINSQRSTNKKVNSHVSFDESTAAPSSKKPGALSAAGGKKSVDDNFSSSVLQTVFALRWQKKAAQKKKKPDDFQQMYMHSMSGALGSIIGDEFDGRKTSGTSSEYGDGEDLEALRILALEKLQAARTRPVAFAVRANYGYNGSEDDDSPVHGMAVSFEKDDCLHIKDKFNKDWWIGRVVKEGHNIGFVPSPSKLKSLQQVGPATGGRPVRGSSKTVFHFNDMVNQAQSPTNTSPSRHSSASVVDAENGMEYNEEEQHSPTSPTSKTSTLPRSASGNTVTSQSAPGQQGKSKKAFFKKQEQLPPYDVVPSMRPIVLVGPSLKGYEVTDMMQKALFDYMKHQFSGRVLISRVTSDISLAKRSNLANPSKRNIIERSNSKNSGLAEVQQEIERIFELSRGLNLVVLDCDTVNHPTQLAKTSLAPLVVYVKISAPKVLQRLIKTRGKTQSRALNVQLVAAEKLAQCSEDLYDLILDETQLQDACHHLGEFLESYWRATHPPNQPGSRPPNMQQSTPQYNVIEAGERPSVYL |
| EdCa_V_β2 with EdCa_V_β1 NTerm | MAQDFALSNRDIELDSLEHVSTGSSTPSEIQRWHMYSDRSGRVVCKDSEPAYRASDTSSVDEDKETSRRELERRAWEALQAARSKPVAFAVRTNIAYEGSEDDDSPVHGAAVSFNVKDFLHVKEKFNDDWWIGRVVKEGCDIGFIPTPSKLKSLQQVGPATGGRPVRGSSKTVFHFNDMVNQAQSPTNTSPSRHSSASVVDAENGMEYNEEEQHSPTSPTSKTSTLPRSASGNTVTSQSAPGQQGKSKKAFFKKQEQLPPYDVVPSMRPIVLVGPSLKGYEVTDMMQKALFDYMKHQFSGRVLISRVTSDISLAKRSNLANPSKRNIIERSNSKNSGLAEVQQEIERIFELSRGLNLVVLDCDTVNHPTQLAKTSLAPLVVYVKISAPKVLQRLIKTRGKTQSRALNVQLVAAEKLAQCSEDLYDLILDETQLQDACHHLGEFLESYWRATHPPNQPGSRPPNMQQSTPQYNVIEAGERPSVYL |
